# Dr. Preston’s Case in the August No.

**Published:** 1890-09

**Authors:** W. S. Gee


### DR. PRESTON'S CASE IN THE AUGUST NUMBER.

EDITORS HOMŒOPATHIC PHYSICIAN:

In last number of your welcome journal an excellent report appeared from the pen of our skilful friend named above, closing with this question: "Have I a right justly to claim that a radical cure has been made?"

After reading the report carefully I would say, No.

From his description I infer that the disease was of the chancroid variety, and while it is questioned by some eminent men whether that form of the disease will produce the full constitutional effect, I have no doubt that this patient ingrafted into his system one of those three great serious miasms.

The subsequent outbreaks of the disease, especially the last two, surely could not have come from a local transfer of the virus, hence we have undoubted evidence that there was a marked constitutional invasion, nor do I believe the Doctor is warranted in saying that his patient is radically free until several years have elapsed, at least three, or he has carried him through some critical illness in which the constitutional miasms within him would be aroused, and no manifestations of the disease appeared. His patient may be free—I hope he is—but I do not think professionally that it is warrantable to declare it a radical cure with the removal of the miasm, but rather that it is probable that the miasm has been quieted by the removal of its manifestations. Time alone will answer it positively. The Doctor is to be much complimented on his skilful management of the case.

Let me ask the Doctor a question: Do you think you ever wholly removed a miasm? If so, how long in doing it?

W. S. GEE.

